# Exposure to Ozone Downregulates Bcl-2 and Increases Executing Caspases-3 and -8 in the Hippocampus, Frontal Cortex, and Cerebellum of Rats

**DOI:** 10.7759/cureus.54546

**Published:** 2024-02-20

**Authors:** Paola Rodríguez-Quintero, Moisés Rubio-Osornio, Eric Uribe, Wilhelm Moreno, Luis A Marín-Castañeda, Zayra Morales, Alonso Portila, David Vázquez, Carmen Rubio

**Affiliations:** 1 Departamento de Neurofisiología, Instituto Nacional de Neurología y Neurocirugía, Mexico City, MEX; 2 Departamento de Neuroquímica, Instituto Nacional de Neurología y Neurocirugía, Mexico City, MEX

**Keywords:** oxidative stress, intrinsic pathway, toxicity, ozone, apoptosis

## Abstract

Introduction

Ozone (O3) is one of the most prevalent atmospheric pollutants, arising from a photochemical reaction between volatile organic compounds (VOC), nitrogen oxides (NOx), and sunlight. O3 triggers oxidative stress, resulting in lipid oxidation, inflammation, alterations in metabolic and cellular signaling, and potentially initiating cell death in vulnerable brain regions. Inflammation and oxidative stress are recognized for their ability to induce cell death, primarily through the apoptosis pathway, involving various proteins that participate in this process via two pathways: intrinsic and extrinsic.

Objective

This study aims to identify the expression of pro-apoptotic proteins and Bcl-2 in the frontal cortex, cerebellum, and hippocampus of rats exposed to O3 acutely.

Methods

Two groups of 20 Wistar rodents (250-300 g) were established. The control group (n=10) was exposed to unrestricted polluted air for 12 hours, while the experimental group (n=10) was exposed to 1 ppm of O3. After exposure, the animals were sacrificed for immunofluorescence and Western blot analysis. Using a t-test, the arbitrary units of pro-apoptotic proteins and Bcl-2 were compared between the two groups.

Results

Significant increases in caspase-8 and caspase-3 activation were found in the O3-exposed group compared to the control group, specifically in the frontal cortex, cerebellum, and hippocampus. Additionally, notable changes in Bcl-2 expression were observed in these brain regions. The TUNEL (terminal deoxynucleotidyl transferase dUTP nick end labeling) assay further indicated significant differences in immunopositivity between the groups in the same areas. However, intrinsic apoptotic proteins such as Bax, VDAC1, and cytochrome-c did not show significant differences between the groups within these structures. Western blot analyses aligned with the immunofluorescence results, showing statistically significant concentrations of caspase-8 in the cerebellum, caspase-3 in the hippocampus, and Bcl-2 in the frontal cortex in the O3 exposed group. Conversely, proteins like Bax, cytochrome-c, and VDAC1 did not exhibit significant differences in all analyzed structures.

Conclusions

This study demonstrates that acute exposure to 1 ppm of ozone can trigger neuronal apoptosis in the frontal cortex, hippocampus, and cerebellum of rats, primarily through the activation of the extrinsic apoptosis pathway via caspase-8 and caspase-3. Additionally, it causes a reduction in Bcl-2 expression, an essential antiapoptotic protein. Despite not observing the activation of intrinsic pathway proteins like BAX, VDAC, or cytochrome-c, the study suggests that chronic O3 exposure might promote cell death by activating this pathway, requiring further long-term research.

## Introduction

Air pollution is a global public health concern that primarily impacts urban areas. It comprises PM2.5, black carbon, nitrogen oxides, carbon monoxide, and other chemical compounds [1]. It is the ninth leading cause of global mortality, contributing to 3.2 million deaths [2]. Ground-level or tropospheric ozone (O3) and PM2.5 pose significant health hazards [3]. O3 is formed through a photochemical reaction involving volatile organic compounds (VOC), nitrogen oxides (NOX), and sunlight. The World Health Organization considers it hazardous when exposure levels exceed >100 g/m3 or 60 ppb on an eight-hour daily average [4]. Ozone is one of the principal tropospheric pollutants and is also among the least controlled; daily exposure exceeds prescribed limits in large cities such as Mexico City, New York, Delhi, and Beijing [3].

The pulmonary inflammatory response and oxidative injury result from an increase in soluble mediators, chemotactic factors, reactive oxygen species (ROS), and reactive nitrogen species (RNS) [5]. Oxidizers such as O3 can overwhelm antioxidant systems, leading to lipoperoxidation, DNA damage, protein deamination, alterations in enzyme activity, and cleavage of peptide bonds. These processes culminate in cell damage that results in necrosis or apoptosis [6]. These effects can also impact the central nervous system (CNS), producing cognitive deficits, neurodegeneration, and loss of brain plasticity in vulnerable regions such as the olfactory bulb, striatum, cerebellum, frontal cortex, and hippocampus. However, specific biological damage pathways remain unidentified [5].

The effects of O3, such as inflammation and oxidative stress, can lead to neuronal apoptosis [7]. This mechanism involves the activation of caspases, resulting in the fragmentation of oligonucleosomal DNA. Any initiator caspase activates caspase-3 and caspase-6, the main caspases that cause apoptosis [8]. Through intrinsic and extrinsic pathways, the cell maintains constant vigilance over its external environment, responding with apoptosis to any detected damage marker [9]. The intrinsic pathway is activated by mitochondrial dysfunction, leading to the release of cytochrome-c into the cytoplasm, which triggers a series of cellular events that culminate in apoptosis [7,8]. The release of cytochrome-c is regulated by a balance between pro-apoptotic (BAX and BAK), anti-apoptotic proteins (Bcl-2, Bcl-xL, and Mcl-1), and molecular sensors including BIM, BID, BAM, Noxa and Puma (BH3) [10]. In the absence of cell survival signals or lesions, such as DNA damage that upregulates p53, BH3 sensors activate and overexpress pro-apoptotic proteins [11]. Thus, BAK/BAX channels and VDAC1 oligomerization generate the mitochondrial permeability transition pore, resulting in the release of cytochrome-c into the cytoplasm and the activation of the initiator caspase-9 [12]. In contrast, the extrinsic pathway involves the activation of specific membrane receptors, including the TNF-1 and Fas receptors or CD95, which leads to the transformation of procaspase-8 into caspase-10 in rodents and caspase-8 in humans. The result of this intracellular signaling is apoptosis [13].

Eventually, both pathways converge into the signaling pathway for the execution phase, triggered by caspases-8, -9, and -10, and activate executioner caspases-3 and -6, and finally, endonucleases that disassemble the nucleus eliminating DNA, RNA, and proteases, which degrade actin and tubulin in the cytoskeleton. Ultimately, cytoplasmic vesicles disassemble the damaged cell into apoptotic bodies [14].

Numerous studies have shown that mitochondrial dysfunction and stress of the endoplasmic reticulum are responsible for the mortality of hippocampal neurons after exposure to 0.25 ppm O3 for 30 and 60 days [15]. However, the mechanisms by which acute inhalation of it is unknown how acute inhalation of O3 can induce CNS apoptosis remain unknown. Consequently, this study investigates the role of caspase-3, caspase-8, Bax, Bcl-2, cytochrome c, and VDAC1 in the initial damage caused by acute exposure to ozone in the frontal brain, cerebellum, and hippocampus of rats.

This article was previously posted to a preprint server (Research Square) on August 28, 2023, and is not currently pending publication elsewhere (https://doi.org/10.21203/rs.3.rs-3290005/v1).

## Materials and methods

In this study, we utilized 20 male Wistar rats weighing 250-300 g as our research subjects because females are more susceptible to O3 and airspace inflammation effects. All efforts were made to minimize the number of animals used and any potential pain or distress. The animals were handled and treated under institutional protocols that complied with national regulations (NOM-062-ZOO-1999) and international guiding principles (Council of International Organizations of Medical Sciences, CIOMS). The rats were individually housed and allowed to move freely in transparent cages with corn-cob bedding at 23±1 °C under a 12-hour light-dark cycle (lights on at 07:00 h). The cages were cleaned once a week and kept dry. 

Rats were randomly divided into two groups (control and experimental) with an n = 10 per group. During the study, subjects were allowed ad libitum access to food and water. The control group was transferred individually to hermetic chambers (30 x 25 x 30 cm) supplied with free air (1.7 l/min) under the same climatic conditions and provided free access to food and water for 12 hours. On the other hand, the experimental group was placed in individual chambers, provided with 1 ppm O3 using a P15 TRIOZON generator (TRIOZON, Tlalnepantla, Mexico). The concentration of O3 concentration was measured and constantly monitored using a Serinus 10 (Ecotech, Melbourne, AU) ultraviolet light analyzer to maintain the 1ppm concentration of O3 constant during 12 hours of exposure (an established parameter that triggers the neuroinflammatory and neurotoxicity response). We exposed three rats per day because we only had three available chambers, which took us two weeks for the experimental phase.

After the ozone exposure phase, the rats were immediately sacrificed using two different techniques: 10 rats (five control and five experimental) by Western blot decapitation and 10 rats (five control and five experimental) by intracardiac perfusion for immunofluorescence. For immunofluorescence procedures, five rats per group were anesthetized with one dose of sodium pentobarbital (50mg/kg). Subsequently, the heart was exposed by making an incision at the level of the xiphoid appendix so that it could be perfused transcardiac with heparin (5000 U/ml), followed by a 300 ml infusion of phosphate buffer saline (PBS) through the right ventricle for 30 seconds and perforation of the right atrial. At the end of the PBS infusion, 4% paraformaldehyde fixation was performed using the same 300 ml volume at a temperature of 4 °C. Subsequently, craniotomy was performed to obtain brain tissue from rats and post-fixed for 24 hours in the same paraformaldehyde fixative at 4 °C. After these procedures, tissue blocks were obtained by embedding paraffin for sagittal slicing in 5 µm slices using a microtome and the Paxinos and Watson rat brain atlas for guidance. The slides were baked overnight at 65 °C, followed by deparaffinization with xylene (30 baths in three stages, five minutes each) and rehydration through graded ethanol (First at 100% and then at 70%) to distilled water. Slides were washed in PBS and incubated for 48 hours at 4 °C.

The tissue was then processed with its corresponding antibodies, all of which were obtained from Santa Cruz Biotechnology, Dallas, TX: Bax (rabbit polyclonal, diluted 1:100, SC-493); cytochrome-c (mouse monoclonal, diluted 1:100, SC-13156), Bcl-2 (mouse monoclonal, diluted 1:100, SC-7382), VDC-A (mouse monoclonal, diluted 1:100, SC-374343), caspase-3 (Alexa Fluor 1:100, SC-7272) and caspase-8 (mouse monoclonal, diluted 1:100, SC-56070) and incubated overnight at 4 °C. After three washes of PBS for five minutes, the appropriate secondary antibody was applied, rhodamine (Rhodamine Red, Anti-rabbit IgG, Jackson ImmunoResearch) for Bax, Rhodamine (Rhodamine Red, Anti-mouse IgG, Jackson ImmunoResearch) for caspase-3 and 8, cytochrome-c and Bcl-2, thus giving the red fluorescence. For VDAC, FITC-conjugated anti-mouse IgG (Jackson ImmunoResearch) was used, producing green fluorescence. Finally, DAPI with Vectashield (ab104139) was added to all slides. These sections were photographed and analyzed with Image-Pro Plus software (Media Cybernetics, Rockville, MD) adapted to an Olympus IX81-F3 microscope (Olympus Corporation, Tokyo, Japan) microscope equipped with a Q-Imaging digital camera kit. The sections were observed with a 40X objective in a field of 520 mm2. The captured areas (Later 1.4 mm) were: Purkinje and granular neurons for cerebellum crus one, hippocampal neurons in Ammon's horn zone 3 or CA3; and neurons in the frontal cortex, all captured in the most apical areas.

Once captures were made, protein densities were quantified using a Q image. We performed a TUNEL (terminal deoxynucleotidyl transferase dUTP nick end labeling) assay using the in situ cell death detection kit, POD (11684795910 Roche), to detect and quantify apoptotic cell death, according to the manufacturer's instructions. The paraffin embedding slices were deparaffinized and rehydrated according to standard protocols. Brain tissue sections were incubated for 30 min at +21 to +37°C with a proteinase K working solution (10-20 μg/mL in 10 mM Tris/HCl, pH 7.4-8). We rinsed the slides twice with PBS, added the TUNEL reaction mixture (50 μL) per sample, and then incubated for 60 min at +37°C in a dark and humidified chamber. After this procedure, the slides were rinsed three times with PBS solution and 50 μL of Converter-POD were added, followed by incubation in a humidified chamber for 30 min at 37 ° C. The slides were then rinsed three times with PBS and 50 μL of DAB substrate was added. Slides were incubated for 10 min at +15 ° C and rinsed three times with PBS. Finally, the slides were analyzed in a drop of PBS under a fluorescence microscope using a wavelength range of 515-565 nm (green). 

The Western blot technique was performed on five rats per group. Subjects were sacrificed by decapitation, extracting the frontal cortex, hippocampus, and cerebellum, placing them in an Eppendorf tube, and keeping them in a freezer at -87°C. The tissues were then homogenized with lysis buffer and protease inhibitor; later, protein quantification by the Lowry method was performed using solution A (NaOH, 2% Na2CO3, 1% CuSO4, 2% sodium potassium tartrate, 10mg/20ml al 0.05% of albumin stock), solution B (Folin's reagent: H2O, 1:1) and bovine serum albumin solution using a spectrometer. For each sample, a buffer cocktail and 2% 2-mercaptoethanol were added. Subsequently, the tissue was heated in a water bath for four minutes; then the samples were refrigerated for later use. Proteins were separated for SDS-polyacrylamide gel electrophoresis (10%) and transferred to nitrocellulose membranes. The membranes were grouped according to their structure and blocked with 5% skim milk in a TBS solution for one hour and incubated with the same antibodies used for immunofluorescence for anti-Bcl2, anti-caspase-3, anti-caspase-8, and anti-β-actin with a concentration of 1:1000 µL per membrane under low shaking at 4 °C. The membranes were washed for four cycles for 15 min each with a TBS-tween solution (250 ml of TBS and 125 µl of tween). After washing, the corresponding secondary antibody was added with a concentration of 1:10000 µL per membrane and incubated for two hours. The washing cycle was then repeated and followed by 1 ml of chemiluminescent solution to visualize and recognize the bands using a photographic plate (Kodak BioMax MR Films, Rochester, NY). Finally, ImageJ software (National Institutes of Health, Bethesda, MD) was used to quantify optical density and compile data using grayscales (8-bit image) and pixels as the measurement unit. We collected the error and standard deviation, gray mean, area, and density intensity for each protein. It is important to note that immunofluorescence and Western blot techniques were performed simultaneously. Therefore, the researchers involved in one process remained unaware of the results obtained in the other procedure until the end of the study.

Statistical analysis 

Pro-apoptotic proteins and Bcl-2 were expressed as mean ± standard error and analyzed using an independent samples t-test for both immunofluorescence and Western blot analysis. Levene's test was initially applied for both techniques to determine whether the variances were equal. As there was an equality of variances, we conducted an independent samples t-test in Microsoft Excel.

## Results

**Figure 1:**
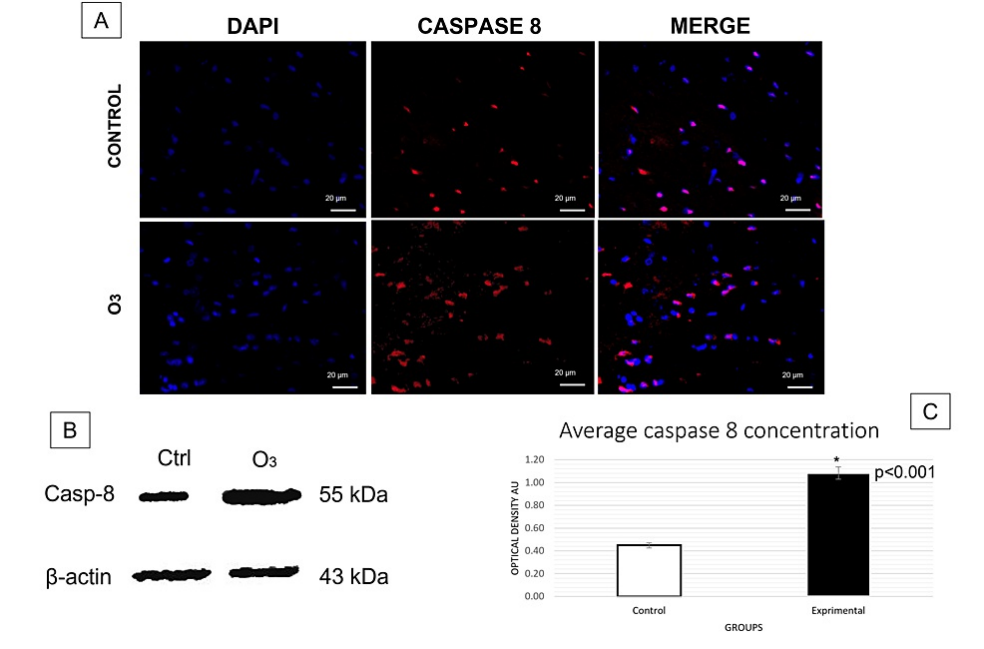
Caspase-8 expression in the frontal cortex. (A) Immunofluorescence was taken in a field of 20 µm with an approach of 40 x (p<0.05). In contrast to the control group, there was an overexpression of caspase-8 in cells of rats of the frontal cortex exposed to acute O3 (12 h). (B) Western blot analysis of β–actin and caspase-8 (p<0.05). (C) The graph bars show the mean number (±2 SE, 95% CI) of arbitrary units of optical density (AU). kDa: kilodaltons

**Figure 2:**
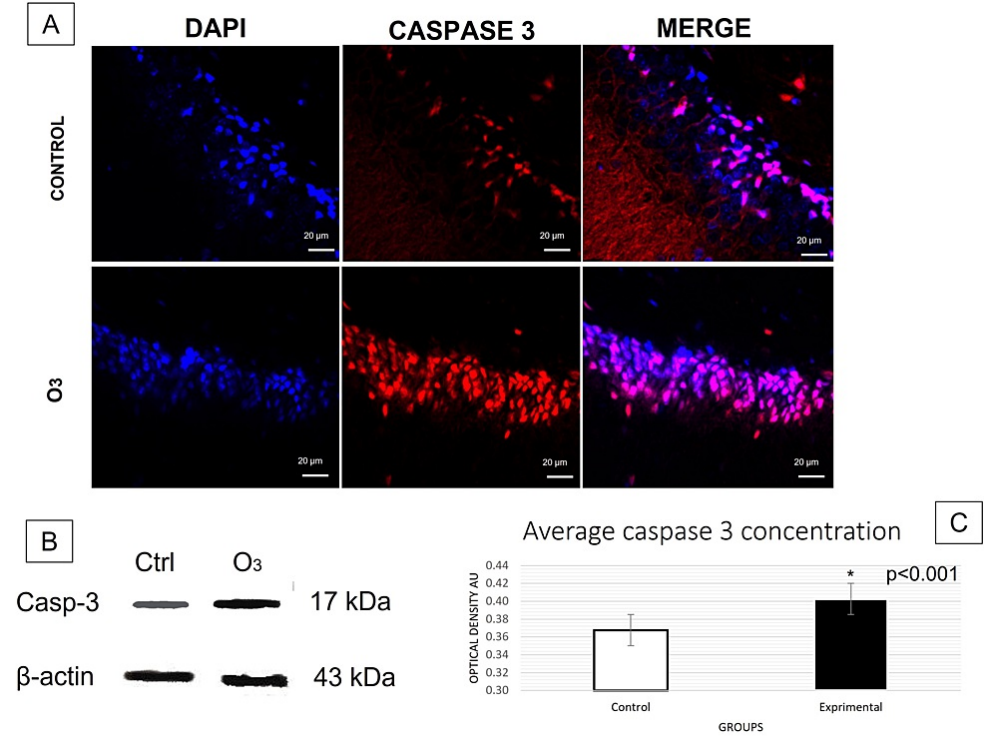
Caspase-3 expression in the hippocampus. (A) Immunofluorescence was taken in a field of 20µm with an approach of 40 x (p<0.05). Note that there were significant differences between groups in both techniques; therefore, acute exposure to O3 increases caspase-3 activity in CA1 of "Cornu Ammonis" of rats. (B) Western blot analysis of β–actin and caspase-3 (p<0.05). (C) The graph bars show the mean number (±2 SE, 95% CI) of arbitrary units of optical density (AU). kDa: kilodaltons.

**Figure 3:**
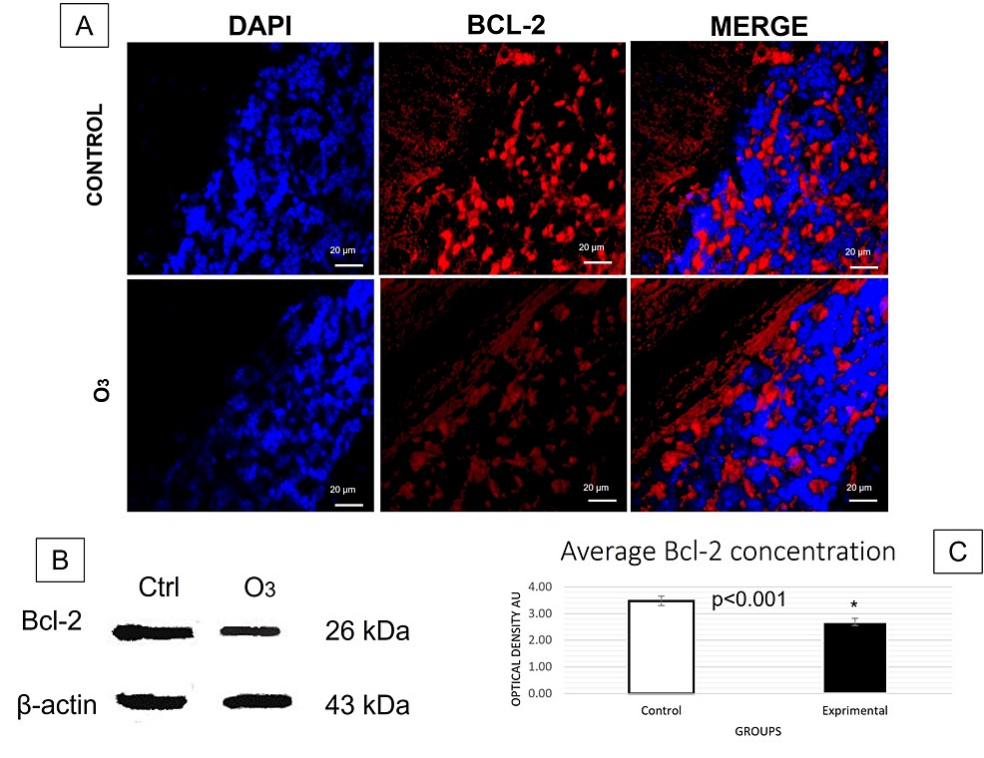
Expression of Bcl-2 in the cerebellum. (A) Immunofluorescence was taken in a field of 20µm with an approach of 40 x (p<0.05). There was a significant difference in the expression of Bcl-2 in cerebellum granular layer cells between the groups. (B) Western blot analysis of β–actin and Bcl-2 (p<0.05). (C) The graph bars show the mean number (±2 SE, 95% CI) of arbitrary units of optical density (AU). kDa: kilodaltons.

**Figure 4:**
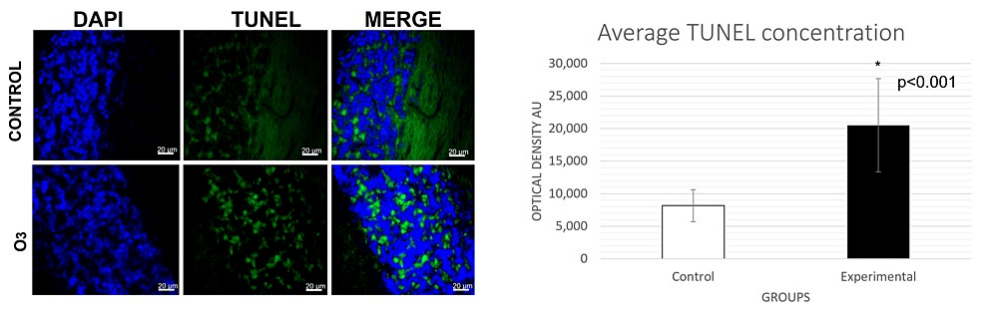
Cerebellum TUNEL assay. Immunofluorescence was taken in a field of 20µm with an approach of 40 x (p<0.05). There were significant differences between groups showing cell positivity in the cerebellum granular layer in the group exposed to ozone. The graph bars indicate the mean number (SE±) of arbitrary units (UA). TUNEL: terminal deoxynucleotidyl transferase dUTP nick end labeling

The t-test conducted for immunofluorescence analyses revealed a significant increase in caspase-8 (Figure 1) and caspase-3 (Figure 2) activation in the group exposed to O3 compared to the control group in the frontal cortex, cerebellum, and hippocampus, respectively. Differences in caspase-3: cerebellum t (31) = 3.457, p = 0.0001, d = 0.6; frontal cortex t (28) = 3.230, p = 0.0015, d = 0.5; hippocampus t (15) = 2.822 p = 0.0064, d= 0.9. Differences in caspase-8: cerebellum t (30) = 5.605, p = 0.0000, d= 0.6; frontal cortex t (27) = 2.963, p = 0.0059, d = 0.5; hippocampus t (12) = 3.186, p = 0.003, d = 1.2. Furthermore, significant changes in Bcl-2 expression (Figure 3) were observed in cerebellum t (29) = 3.456, p = 0.0008, d= 0.53; frontal cortex t (29) = 3.548, p = 0.0006, d = 0.7; hippocampus t (18) = 3.593 p = 0.001, d = 0.7. Furthermore, the TUNEL assay (Figure 4) showed significant differences in immunopositivity between the groups: cerebellum t (31) = 3.026, p = 0.0024, d = 0.8; frontal cortex t (27) = 6.042, p = 0.0000, d = 1.2; hippocampus t (13) = 2.695, p = 0.009, d = 1.3. However, intrinsic apoptotic proteins such as Bax, VDAC1, and cytochrome-c did not have significant differences or effect sizes between both groups within the three analyzed structures. Furthermore, the most representative images are shown in this article and the statistical significance of the results has been discussed previously. 

The t-test in the Western blot protein bands analyses yielded results similar to the immunofluorescence results. Caspase-8 in the cerebellum t (3) = 2.857, p = 0.03, caspase-3 in the hippocampus t (3) = 3.568, p= 0.001, and Bcl-2 in frontal cortex t (3), p=0.0009, exhibited a statistically significant higher concentration in the O_3_ exposed group compared to the control group. However, the remaining proteins analyzed, such as Bax, cytochrome-c, and VDAC1, did not show statistically significant differences in all structures.

## Discussion

According to the findings, short-term exposure to 1 ppm of ozone resulted in increased levels of both initiator caspase-8 and executor caspase-3 in structures such as the hippocampus, cerebellum, and frontal cortex of rats. Caspase-3 by itself does not differentiate whether the activated apoptotic pathway is extrinsic or intrinsic since it can be started by caspase-8 or -9, respectively. With a discernible trend of caspase-8 overexpression in the experimental group, we can confirm that at least the extrinsic apoptotic pathway is activated.

**Figure 5:**
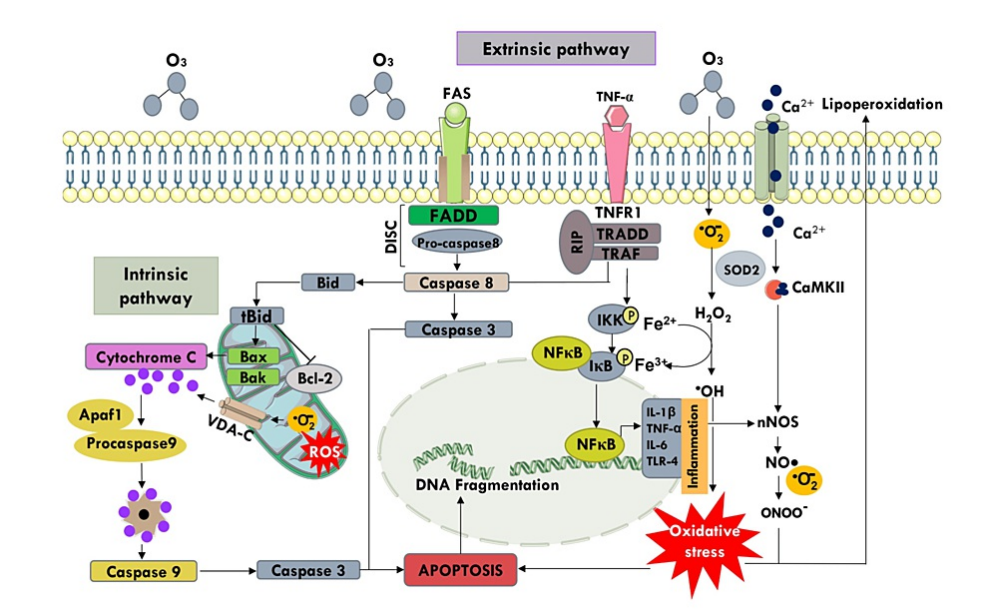
Extrinsic and intrinsic apoptosis pathways. Extrinsic apoptosis is induced by death receptor ligands such as FAS or TNF. They bind to their receptor TRADD or FADD and then to procaspase-8, forming DISC that activates caspase-8, which triggers apoptosis that activates caspase-3, resulting in neuronal death. The intrinsic pathway triggers due to the oxidative stress generated by the excessive production of ROS, the saturation of antioxidant systems, aerobic metabolism, and the activation of the calmodulin pathway forming superoxide anion (•O2-), hydrogen peroxide (H2O2), hydroxyl radical (•OH) and peroxynitrite (ONOO-), due to the entry of excessive Ca2+ which also stimulates the phosphorylation of the IκB resulting in the upstream of Nf-κB which activates the transcription of cytokines and other pro-inflammatory markers that lead to chronic oxidative stress, compromising cell function, resulting in neuronal death. TNF: Tumor necrosis factor; TRADD: TNFR1-associated death domain protein; FADD: Fas-associating protein with death domain; DISC: death-inducing signaling complex; ROS: reactive oxygen species; IκB: kappa-N inhibitor. This image was created by our research team specifically for this study.

This study highlights the prevalence of the extrinsic pathway as the primary driver of cell death (Figure 5). This observation is based on the absence of significant expression of pro-apoptotic proteins associated with the intrinsic pathway, including BAX, VDAC1, and cytochrome-c, within these structures. Several factors may explain this molecular behavior; one of the reasons could be that the harmful central stimulus for ozone intoxication arises from the cell's external environment. 

**Figure 6:**
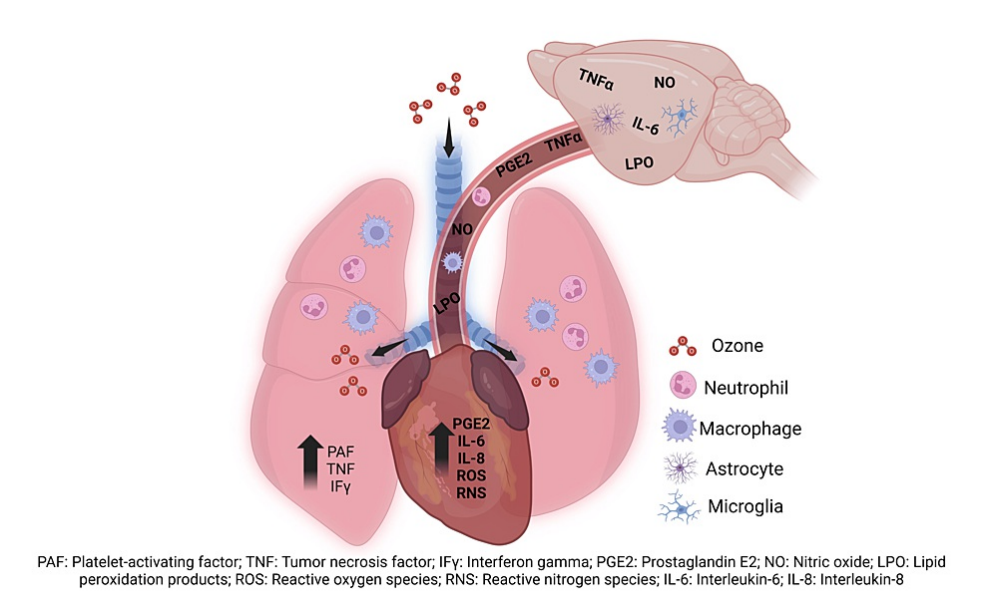
Passage of ozone oxidation products to the brain via systemic circulation. Exposure to ozone triggers the activation and release of cellular oxidation-related products and the activation of immune cells. Cells such as macrophages, neutrophils, and pro-inflammatory cytokines can travel to other tissues, such as the brain, through systemic circulation. Once in the brain, they will activate inflammatory and cell death processes as well as promote reactive gliosis. PAF: platelet-activating factor; TNF: tumor necrosis factor; IFy: interferon-gamma; PGE2: prostaglandin E2; NO: nitric oxide; LPO: lipid peroxidation products; ROS: reactive oxygen species; RNS: reactive nitrogen species; IL-6: interleukin- 6; IL-8: interleukin 8 This image was created by our research team specifically for this study.

It is worth noting that ozone-exposure-induced intoxication can trigger a cascade of pro-inflammatory cytokines within the lungs, leading to the activation of immune cells. This unremitting pro-inflammatory cascade continues into the systemic circulation, potentially reaching other tissues, including the brain. Once it reaches the brain, the blood-brain barrier primarily undergoes endothelial damage, which subsequently allows the passage of pro-inflammatory products into the brain parenchyma (Figure 6). One of these products is TNF-α, a pivotal activator of cell death receptors, making it a potential key marker of damage in hypoxia-vulnerable structures such as the frontal cortex, hippocampus, and cerebellum. Another plausible reason for the limited involvement of the intrinsic pathway during acute O3 exposure is the temporal aspect of its activity. Overheating of these systems cannot activate the intrinsic pathway because redox homeostasis is maintained, and antioxidant systems like Nrf2/ARE, SOD, and GST continue to function during acute exposure to O3 [16].

Several reports [17-19] mention that the antioxidant activity of the Nrf2 pathway in response to low doses of O3 is found after three to six weeks of exposure, initially through an increase in the activity of its receptor protein Keep 1 (cytosolic inhibitor). Cytochrome-c, an essential one-electron carrier, determines its fate based on its redox state, whether it exists in a reduced state, carrying an electron, or an oxidized state, devoid of an electron. Therefore, the apparent lack of activity of cytochrome-c within the experimental cohort can be attributed to its association with the oxidative state in which it is found; it must be oxidated to activate caspase-9, thus undergoing apoptosis through the intrinsic pathway. However, though cytochrome-c could be reduced into the cytosol once released to maintain the cytosolic reserve against efflux, investigations showed that there are cytochrome-c reducers such as ascorbate, superoxide, glutathione, NOS, neuroglobin, and cytochrome p450 reductase. In the context of ozone exposure, the reduction reaction accelerates, rapidly lowering the cytochrome-c cytosolic concentration [20]. In other words, we might observe a neuroprotective effect on the antioxidant enzymatic machinery that could impede the progression of apoptosis through the intrinsic pathway.

In terms of the BAX protein, previous research shows a discrepancy in space-time activation of BAX and BAK due to their close relationship with another member of the Bcl-2 family, Bcl-xL. This intricate dynamic occurs in the relocation of BAX to the cytosol, triggering a regulatory process intended to stabilize the apoptotic machinery in an inert state [21]. This could explain why it is not overexpressed in the experimental group. In contrast, the precise role of VDACs in apoptosis remains poorly understood, promoting debate in the scientific community on whether this protein opens or closes its channels during apoptosis [12,22]. There are three theories regarding VDAC's role; the first and the second lead to cytochrome-c release, where VDAC induces the opening of mitochondrial external membrane channels, and homo/hetero oligomerization promotes pore enlargement [23,24]. The third theory, which aligns with our findings, is the only one that defends that VDAC has a closure mechanism in apoptosis that leads to a high metabolite concentration that interferes with an increase in mitochondrial volume. This eventuality precipitates the breakdown of the external membrane, culminating in the release of cytochrome c into the cytosol [25]. 

Although the intrinsic apoptotic pathway does not appear to be active in our results, the anti-apoptotic protein Bcl-2, which directly participates in this pathway by preventing the formation of the mitochondrial permeability transition pore and the release of cytochrome-c into the cytosol, is decreased in the experimental group across all three areas of the brain analyzed. This suggests that acute exposure to O3 has an additional antiprotective effect, decreasing the probability of cell survival. This result reinforces the hypothesis that, although we did not observe pro-apoptotic activity in the intrinsic pathway in rats exposed to ozone, the protective effect exerted by Bcl-2 could eventually lead to mitochondrial dysfunction with chronic exposure to ozone.

Furthermore, the results of the TUNEL assay were significantly positive for the O3 group. This assay exposes one of the molecular characteristics of apoptosis: nuclear DNA fragmentation, facilitated by nucleases. Our result confirms the participation of caspases, as they are known to activate nucleases in the execution phase. Furthermore, these results support the clinical implications of O3 exposure. It is important to note that apoptosis plays an essential role in the development of various neurological pathophysiological mechanisms, especially in neurodegenerative diseases. In these neurological disorders, pro-inflammatory markers such as TNF-α, IL-1β, IL-2, INFγ, NOS, and ROS are increased according to postmortem brain samples. These markers are recognized to be involved in programmed cell death due to the pro-oxidant effects that O3 exerts, making its exposure a significant risk factor for disease progression and development [26]. 

Several studies establish a link between ROS production due to O3 and subsequent disruptions in enzymatic function, lipoperoxidation, and protein oxidation, culminating in the loss of primary and secondary dendritic spines, provoking neurodegeneration [27]. Endoplasmic reticulum (ER) dysregulation and hippocampus stress are two mechanisms of cell damage in other acute and chronic neural diseases. Chronic exposure to O3 can mimic this mechanism by increasing intracellular calcium concentration and activating the ATF6 pathway due to chronic oxidative stress, primarily due to increased ER calcium by TNF-α [15]. The ATF6 pathway regulates cell death, and when this system suffers a critical breakdown, Ca2+ enters the ER and activates the apoptotic pathway through caspase induction [28].

Similarly, the cerebellum has been observed to be a structure significantly affected by exposure to O3. In newborn rats, the dynamics of neurotransmitters such as GABA and glutamate modulation in Purkinje cells are altered [29]. These can lead to motor impairments such as ataxia and limited cortical brain recovery after damage. Furthermore, in rats exposed to ozone for five days, a significant increase in TNF-α and IL-6 levels was observed in the frontal cortex after only six hours of exposure. These cytokines induce an inflammatory response in the CNS with activation of apoptosis [30].

Furthermore, the realm of sleep has been investigated in the context of ozone intoxication in rats. Studies have shown that exposures ranging from 0.5-1.0 ppm of O3 can suppress rapid eye movement (REM) sleep and increase non-REM patterns, mainly due to decreased concentrations of acetylcholine in the hippocampus and increased concentrations of serotonin and dopamine in the brain stem [5].

## Conclusions

Atmospheric changes due to excessive human activity pose significant contemporary challenges, notably impacting public health. Among the primary pollutants in large cities, tropospheric ozone stands out for its pro-oxidant effects on pulmonary health, with studies extensively documenting its impact. Yet, the mechanisms through which ozone causes damage to other tissues remain largely unexplored. This gap in knowledge underscores the need for further investigation into the broader implications of ozone exposure on human health.

The current study reveals that acute exposure to ozone triggers the activation of neuronal apoptotic machinery in the frontal cortex, hippocampus, and cerebellum. This apoptotic activation is primarily attributed to the extrinsic apoptosis pathway, as evidenced by the activation of caspase-8 and caspase-3, and is further characterized by a reduction in Bcl-2 expression, a critical antiapoptotic protein within the intrinsic pathway. Despite the absence of pro-apoptotic proteins associated with the intrinsic pathway, such as BAX, VDAC, or cytochrome-c, the study posits that chronic oxidative stress from ozone exposure may ultimately promote cell death through this pathway. There is a need for long-term studies to validate this hypothesis and explore the potential link between ozone exposure and the onset of neurological disorders.
